# Evaluation of the Emotional and Cognitive Regulation of Young People in a Lockdown Situation Due to the Covid-19 Pandemic

**DOI:** 10.3389/fpsyg.2020.565503

**Published:** 2020-10-23

**Authors:** Manuel Fernández Cruz, José Álvarez Rodríguez, Inmaculada Ávalos Ruiz, Mercedes Cuevas López, Claudia de Barros Camargo, Francisco Díaz Rosas, Esther González Castellón, Daniel González González, Antonio Hernández Fernández, Pilar Ibáñez Cubillas, Emilio Jesús Lizarte Simón

**Affiliations:** ^1^Department of Didactics and School Organization, Faculty of Education Sciences, University of Granada, Granada, Spain; ^2^Department of Pedagogy, Faculty of Education Sciences, University of Granada, Granada, Spain; ^3^Department of Didactics and School Organization, Faculty of Education, Economy and Technology, University of Granada, Ceuta, Spain; ^4^Department of Research Methods and Diagnosis in Education, Faculty of Education and Sport Science, University of Granada, Melilla, Spain; ^5^Department of Research Methods and Diagnosis in Education, Faculty of Education Sciences, University of Granada, Granada, Spain; ^6^Department of Pedagogy, Faculty of Humanities and Education Sciences, University of Jaén, Jaén, Spain

**Keywords:** emotional cognitive regulation, higher education, dropout, pandemic covid-19, lockdown

## Abstract

**Background:**

In a situation of compulsory home isolation enacted by governments at the spreading of the Covid-19 pandemic, the emotional health and well-being of students became a key factor in the successful implementation of distance teaching methodologies in face-to-face education universities. Psychological well-being, an essential factor in preventing academic failure, has been threatened in this serious situation of unprecedented and stressful isolation. The aim of this study is to analyze the students’ cognitive-emotional regulation as well as their beliefs and perceptions about the pandemic and this lockdown situation. With this extensive study we are carrying out, want to describe the extent to which the lockdown situation is a risk factor, and, in the future, make proposals for preventive and palliative actions, if necessary, to minimize this potential risk.

**Method:**

We applied the CERQ Cognitive Emotion Regulation Questionnaire by means of an online application together with a questionnaire, CC/covid-19, of objective description and subjective perception of the lockdown situation of the students, their conditions to study, general opinions about the pandemic and specific opinions about the real possibilities of implementing online education in the middle of the academic year at the university. 1910 valid responses from more than 80 universities in 13 different Spanish-speaking countries were obtained and submitted to descriptive analysis and modeling using structural equations.

**Results:**

Most of them consider that the lockdown decision is correct, that health systems are not prepared to deal with the pandemic, and that although the universities have adequate means, the teaching staff is not competent to implement online teaching methodologies. They have a good perception of the conditions of isolation, however, the time devoted to studying has not increased. One of the results of our study is the students’ self-evaluation about their digital competence and their capacity to perform in online interactive communication. This is key to rejecting a feeling of loneliness or social isolation, even if there is momentary physical separation with friends and classmates which is consistent with the results of emotional well-being the surveyed students present. The cognitive strategies used by the students surveyed have allowed them coping with events arising from the pandemic, mandatory isolation and university closure, certainly adaptive and functional, while maintaining a positive perception of their new living and learning situation.

## Introduction

We know emotions guide response behavior to external stimuli as an adaptive reaction of people in their daily lives. Behavior does not always have to be controlled, but in those situations where it must be adapted, emotional self-regulation is necessary. The main strategies of emotional regulation are intentional deployment, cognitive re-evaluation and response modulation (Gross, 2015), and there seems to be an agreement in that the most frequent and adaptive strategy of emotional regulation is precisely cognitive re-evaluation (McRae, 2016). This re-evaluation makes possible to introduce changes in the way a situation is perceived in order to change the way it is felt, ultimately exerting control over the feeling itself and its intensity: maintaining, increasing or suppressing it.

The process of cognitive emotional regulation can be difficult for some people, especially after they have experienced particularly stressful events, to the point where a vicious circle is established (Stikkelbroek et al., 2016). The cycle is fuelled by information processing biased toward negative self-thoughts and by cognitive assessment difficulties. This is a problem for emotional regulation. These stressful events generate symptoms of depression.

For the general population, academic stress does not generate such important pathologies. It is precisely in the educational context where the processes of emotional regulation are tested early on. However, it is clear that academic stress influences students, that it does it differently in each person, and that emotional regulation, through cognitive assessment, allows them to adapt their behavior in accordance with perceived goals. Emotional regulation influences the cognitive processes involved in learning, from motivation, to the processing of information, to the establishment of significant links between new content and previous knowledge, and, of course, to the use of information or the demonstration of what has been learned through tests and exams (Fernández, 2015). This is why it is so important for educators and teachers to know the quality of cognitive assessment students carry out of the emotions in stressful academic situations and its effect on academic performance. This knowledge will be at the base of the prevention of academic failure.

There is extensive literature on the relationship between coping styles and strategies and academic performance (Ávila Quiñones et al., 2014). Coping is a response to stressful situations that can threaten a student’s academic progress. Coping can be adaptive and related to positive variables of psychological well-being such as self-regulation (Morales and Trianes, 2010) or, on the contrary, passive, maladaptive, paralyzing and generating dissatisfaction and stress. The student who lives a disadaptive coping is at risk of academic failure and drop-out. As pointed out by Tejedor et al. (2007), even when failure has a multifactorial origin, teacher training to know and guide the coping strategies of students at risk, can be a good preventive practice. Ávila Quiñones et al. (2014) insist on demanding the improvement of academic guidance processes, the teaching of study techniques and habits and the training of attitudes of responsibility, effort and self-demand as preventive measures.

Between March and April 2020, a large number of governments in Western Europe and Latin America, among many others around the world, took measures to restrict the movement of people to deal with the Covid-19 pandemic that had started 5 months earlier in Wuhan a province of China. Among the restrictive measures taken were the closure of universities and the declaration of lockdown. In this lockdown situation, universities continued to operate and adapted, in a rapid and compulsory manner, face-to-face teaching to distance education based on the use of online resources and telematic means. Two months later, lockdown and university closure were still in place.

In this new situation, the mental health and general psychological well-being of students became an essential key factor for the success of the new education modality based on the implementation of distance learning methodologies and resources (Shea and Bidjerano, 2019).

We have previously studied how the academic failure of young students in Higher Education, and its worst consequence – dropout – has serious personal, social and economic effects (Aulck et al., 2017). We know that early diagnosis of causes and risk groups can minimize the severity of these effects as sought by academic and policy makers (Mortagy et al., 2018). This is a complex and multi-causal phenomenon that can be explained by causes related to the personality of the youth; to the available structures of social integration; to the investment in time, money and effort that the student must make to complete his or her studies; to the institutional arrangements for hosting and accompaniment; to the effectiveness of the instructional model offered; and to vocational aspects (Lizarte, 2017).

Among the causes of psychological order, we can mention the deficient emotional regulation of young people and its consequent difficulty to face stressful academic situations such as the effort to study, the accomplishment of activities within a set period of time, the demonstration of their knowledge in front of the evaluator or taking exams. To cope with this situation, scholars call for social support, institutional support, and personal commitment (Bar-Am and Arar, 2017); personal commitment requires general psychological well-being.

In short, psychological well-being, which is an essential factor in preventing academic failure, was threatened in this serious situation of unprecedented and stressful isolation. Therefore, it seemed necessary to investigate this issue with the following objective: to evaluate the cognitive emotional regulation of students as well as to reveal some of their beliefs and perceptions about the pandemic and the lockdown situation.

We know that a situation of pandemic and lockdown causes feelings of anxiety and fear in the whole population (Singh et al., 2020). This includes the youth population. We also know that the feelings of anxiety and fear that may have, in some subjects, undesirable consequences on their mental health, depend on previous psychological vulnerability and other pre-existing social, cultural, and economic conditions of vulnerability, as well as on the specific environmental conditions in which the lockdown takes place (Judge and Rahman, 2020). In a situation of lockdown, the development of feelings of anxiety among students (Majumdar et al., 2020) is influenced by general beliefs about the pandemic, beliefs about the effectiveness of the new on-line education situation, social conditions and domestic coexistence, as well as housing conditions.

With the extensive study we are carrying out, in a first instance, we want to describe the extent to which the lockdown situation is a risk factor, and, in the second instance, to make proposals for preventive and palliative actions, if necessary, to minimize that potential risk (Bolhaar et al., 2019). Proposals valid for the current situation of distance learning but also valid when face-to-face teaching has returned to our classrooms.

## Materials and Methods

### Design

To evaluate the cognitive emotional regulation of students, a diagnostic instrument validated in populations of adolescents, young people and university students both in Spain and in different regions of the world was selected. It is the Cognitive Emotional Regulation Questionnaire (Garnefski et al., 2001; Garnefski and Kraaij, 2007) which is used to study how cognitive coping works in difficult, threatening, stressful or traumatic situations, through different strategies.

The instrument can be used to assess the general response to stress, or to assess responses to specific events. In our case, the instrument was used as a questionnaire to assess specific coping in the situation of forced isolation and university closure.

This instrument was applied by means of an online application together with a questionnaire of objective description and subjective perception of the lockdown situation of the students, their conditions to study, general opinions about the pandemic and specific opinions about the real possibilities of implementing online education in the middle of the academic year at the university. This instrument, CC/covid-19, was prepared specifically for this study.

The study has been conducted in four phases:

(1) *National focus*. During the first phase, the instruments have been applied on a national scale using contact networks of university teachers from all public and private universities in Spain. The implementation started during the second week of isolation in Spain during 4 weeks. The aim of this phase was to study emotional regulation in different personal situations and with different perceptions of the same national situation of isolation.
(2) *International extension*. In the second phase, the study was extended to 13 Latin American countries as their governments implemented university closure and similar isolation measures, but never so strict like those in Spain. The Latin American region was chosen to apply the questionnaires in the same language: Spanish. With this international expansion we intended to contrast the study focused on Spanish youth. This international extension was carried out during the month of April 2020.
(3) *Contrast in Second Wave*. In a third phase, a second wave of the instruments has been applied to the same national and international population that has participated in the previous phases. The aim is to find out how the use of different coping strategies has evolved, as well as the description and perception of their personal situation by the young people surveyed. This contrast operation may be repeated if the measures implemented by the spread of the pandemic are maintained or reappear over the time. Each contrast can be compared with the baseline already established.
(4) *Elevation of proposals and recommendations*. In the last phase we intend to draw up proposals and recommendations to the political and university authorities with measures for the prevention of academic failure among university students.

### Sample

The questionnaire was applied via a convenience and snowball sampling (Rockinson-Szapkiw et al., 2019) and distributed through different networks of university professors who, interested in the study, were willing to send it to their students requesting their collaboration. The application in Spanish universities began on March 2020 and was extended to Latin American universities in the way different governments of the region started to implement measures to close universities and confine students to their homes. Mexican universities were the last ones to join the study. We closed the first two phases of the research 6 weeks after its beginning, obtaining 1910 valid responses for the analysis.

The sample obtained is made up of 1464 women (76.6%), 434 men (22.7%), and 12 individuals who identify their gender as “other” (0.6%). The age of the participants ranges from 18 to 37 years, with 21 years being the mode age for 14% of the young people surveyed. In addition to Spain (which represents 81.9% of the sample) the participants reside in 13 other Latin American countries of which Mexico is the most represented with 9.7%. The students belong to 81 different Higher Education Institutions, with the Spanish University of Las Palmas de Gran Canaria with 30.5% and the University of Granada with 23.6% being the most represented. The sum of both universities represents more than half of the sample.

We found out that 791 respondents are studying a degree or diploma in Educational Sciences, i.e., 42% of the total. The remaining respondents are widely spread among other professional and scientific fields. Meanwhile, the level of the students is homogeneously distributed among the different years of study: 26.9% in the first year, 20.2% in the second, 18.3% in the third, and 20.4% in the fourth year of study.

### Instruments

#### CERQ

The Cognitive Emotional Regulation Questionnaire is an instrument of 36 items grouped into nine dimensions referred to other cognitive strategies of emotional regulation. Five of them help to reduce the unpleasant state caused by the stressful experience, such as Acceptance, Putting into Perspective, Positive Refocusing, Positive Reappraisal or Refocus on Planning, while the other four influence the increase of the unpleasant state, such as Rumination, Catastrophizing, Self-blame, and Other-blame.

Acceptance is the cognitive process of trying to live the stressful situation without generating negative emotions. Putting into Perspective allows us to put the stressful event into perspective by comparing it to others in order to relativize its severity. Positive Refocusing consists of directing attention to pleasant thoughts that diminish the effect of the stressful situation. Positive Refocusing requires not focusing exclusively on the negative consequences of an event, but seeking out its fewer negative aspects. Refocus on Planning allows us to focus our thoughts on solving the problem.

Rumination consists on focusing thoughts on the seriousness of the event and its possible consequences. Catastrophizing is the anticipation of disproportionate and extremely serious consequences. Self-blame is the personal assumption of the cause of the stressful event. Other-blame is the obsessive attribution of the cause to the action of others.

All the nine strategies can be functional and adaptive at any given time.

The response to the items in each dimension is made by means of a 5-point Likert scale, in which (1) indicates low frequency and (5) high frequency. In an individual the score in each dimension can range from 4 to 20 so that the scores obtained in the dimensions show the frequency of use of each of the cognitive strategies involved.

For its application, the Spanish version of the questionnaire translated and validated by Domínguez-Sánchez et al. (2013), was used. It was adapted specifically for this situation of mandatory isolation and university closure.

#### Lockdown Questionnaire CC/Covid-19

A specific Lockdown Description and Perception questionnaire was developed to be applied in conjunction with the CERQ. This allowed us to study possible relations between the personal situation of isolation and the use of different cognitive strategies of emotional regulation. The questionnaire CC/covid-19 is made up of six sections and a total amount of 41 items:

(a) Sociodemographic data that allowed us to describe the sample. It consists of six open response questions.
(b) Inventory of general beliefs about the covid-19 pandemic. It consists of five response questions on a 5-point Likert scale. General beliefs about the pandemic include issues related to their perception of the health measures taken by the authorities. It is not a question to extract from them an expert or professional opinion that they do not have, but to know the position that they take as ordinary citizens of the situation.
(c) Inventory of general beliefs about the effectiveness of on-line education. It consists of five response questions on a 5-point Likert scale.
(d) Social conditions of the lockdown and description of the coexistence situation. It consists of one open question and seven dichotomous answer questions.
(e) Habitability conditions and description of the place of Isola. It consists of 10 questions with a dichotomous answer.
(f) Description and perception of the living situation in the place of isolation. It consists of eight questions with a dichotomous answer.

The construction of the questionnaire was carried out in three phases. In the first phase the relevant dimensions were selected for the description of the lockdown. In the second phase, a literature review was conducted for each dimension to extract the issues that were considered most relevant. In a third and final phase the list of items of each dimension was readjusted and refined with the idea of representing each of them with the minimum number of questions so that the instrument would not be that long.

The selection of questions for each section was entrusted to a different expert from the University of Granada Profesio-Lab Research Group, who was responsible for the study. This selection was submitted to discussion by the plenary of the Group until the adjustments made was considered valid.

## Results

### Descriptive Analysis CC/Covid-19

Table 1 shows the results obtained on general beliefs about the pandemic. Students strongly agree that older people can spread the virus (4.13) and that they themselves are agents transmitting the virus to the older population (3.95). They disagree with the statement that authorities have taken the right measures (2.74), that the national health system (in each reference country) is prepared for a pandemic (1.97) and, finally, they strongly disagree with the idea that they are immune to the virus (1.45).

**TABLE 1:** Beliefs about the Covid-19 pandemic.

**Belief**	***N***	**$\bar{X}$**	**DS**
Young people are virus transmitting agents to the elderly	1910	3.95	1.028
Young people are immune to the virus	1910	1.45	0.695
Older people can infect me the virus	1910	4.13	0.917
The authorities have been able to take appropriate action	1910	2.74	1.111
National Health System is prepared for pandemic	1910	1.97	0.936

In Table 2 you can see their beliefs about the effectiveness of the online education modality implemented after the university closure. The majority of the young people interviewed agree they have the necessary digital competence to be able to perform well in online education (3.76). They also agree that teachers have the necessary resources for this type of teaching (3.02). On the remaining issues there is more disagreement than agreement. Below average is the consideration that the university has adequate services and resources for this type of teaching (2.95) and some (2.93) belief that during lockdown the study time is better planned. Where there is less agreement (2.47) is in the belief that teachers are prepared for online teaching.

**TABLE 2:** Beliefs in the effectiveness of online learning.

**Belief**	***N***	**$\bar{X}$**	**DS**
My university has the appropriate services and resources for e-learning	1910	2.95	1.156
Teachers are prepared to develop online teaching	1910	2.47	1.119
Teachers have the necessary resources to develop online teaching	1910	3.02	1.120
I am digitally competent to manage in online education	1910	3.76	1.062
I improve the study time planning during lockdown	1910	2.93	1.274

In Table 3 we have collected data related to the social conditions of isolation and the description of the living situation. The average number of people in lockdown situation is between 3 and 4 (3.58), with the minimum being 1 (the student in isolation and solitude), and the maximum 13. The great majority of students (83.4%) have been confined to the family residence and do not live neither with people over 70 years of age (a special population at risk) nor with pets that require a walk thus eventual socialization.

**TABLE 3:** Lockdown social conditions and description of the living situation.

	***N***	**Min**	**Max**	**$\bar{X}$**	**DS**
Number of people confined at home	1868	1	13	3.58	1.419
			**Yes %**	**No %**	***N***
Family residence			83.4	14.2	1864
Any other relatives house			7.4	92.4	1905
Students eventual home			24.6	74.7	1895
Live together with kids			22.5	76.9	1897
Live together with adults older tan 70 years old			13.7	86.0	1903
Live together with pets that require being taken for a walk			34.0	64.6	1883

In Table 4, we included data describing the house, which allowed us to indicate that most of the respondents have been confined to a dwelling located in an urban setting (74.1%) that mostly lacked a garden, terrace or patio, but at least had exterior views (85.1%).

**TABLE 4:** Description of the place of isolation.

	**Yes %**	**No %**	***N***
Urban housing	74.1	23.8	1870
Rural housing	22.9	76.1	1890
House with large garden	16.5	82.9	1898
House with small garden	13.6	86.2	1906
House with large terrace	28.1	71.3	1896
House with small terrace	32.2	66.6	1886
House with a large interior courtyard	16.0	83.5	1900
House with small interior courtyard	24.2	75.5	1904
House without any outside space	23.2	76.2	1899
House with views	85.1	12.2	1859

In Table 5 we find data referring to the perception of housing suitability. We can see that most of them qualify their house as spacious (60.8%), comfortable (88.6%), with wifi connection (92.6%), well equipped to study (77.6%), and good equipment for leisure (64.3%). Only one negative qualification stands out; it refers to the absence of equipment for physical exercise, indicated by more than half of population surveyed (52.6%). Although in a low percentage (4.6%) we are concerned about the fact that there are students who lack Wifi connection which deprives them from following distance learning, and what is worse of keeping fluid social contact through social networks. However, we think that the widespread use of mobile phones alleviates the difficulty of social interaction as we have already pointed out in previous studies (Martínez-Sánchez et al., 2020).

**TABLE 5:** Perception of housing adequacy.

	**Yes %**	**No %**	***N***
Large house	60.8	37.5	1878
Comfortable housing	88.6	9.1	1865
Small house	30.4	68.5	1889
Cramped housing	12.6	86.8	1897
WiFi connection	92.6	4.6	1857
Housing with good studying facilities	77.6	20.3	1870
Housing with good equipment for physical exercise	46.3	52.6	1889
Housing with good leisure facilities	64.3	33.6	1869

### Descriptive Analysis CERQ

**FIGURE 1:**
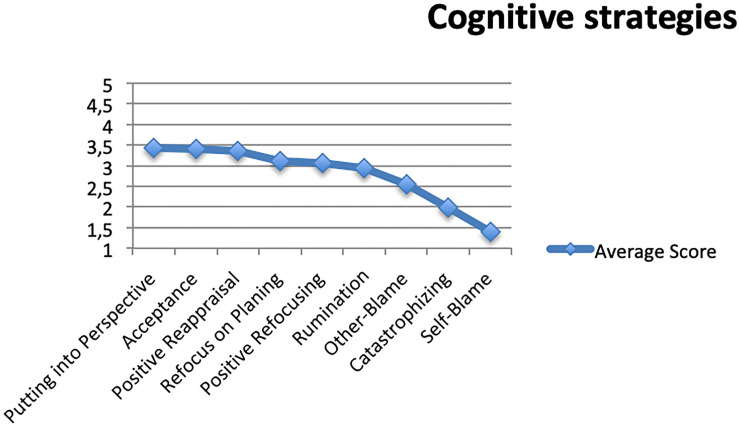
Frequency of use of cognitive strategies.

In Figure 1 we include the frequency of use of each of the nine cognitive strategies analyzed using the arithmetic mean of the score obtained in each of the four items that make up each dimension or strategy. In the figure we have ordered the scores from highest to lowest frequency. Five of the strategies have a score above the average. These are precisely the ones that are characterized as more functional and adaptive insofar as they tend to diminish the effect of the negative feelings associated with the isolation situation. The most widely used is Putting into Perspective, with a score of 3.43, in which students put into perspective compulsory isolation by comparing it to more serious or stressful events. It is often followed by the Acceptance strategy with a score of 3.40, which allows students to deal with the lockdown without judging it and, therefore, generating negative feelings. The third strategy is that of Positive Reappraisal with 3.36 points, whereby the young people try to focus on those positive aspects that may be associated with the lockdown situation. The following in frequency is Refocus on Planning with 3.11, leading students to think about action plans and problem solving as a coping strategy. Positive Refocusing, with 3.07, leading students to redirect their attention to pleasant thoughts as a way of coping with the problem is the next one. These six cognitive strategies get above-average scores in each dimension. They are the ones most frequently used by young people.

Rumination, which defines coping as strongly focusing thoughts on the stressful situation lockdown represents, scores below the arithmetic mean, 2.94. It is followed by the strategy of Other-blame to face the current situation with 2.55. Much less frequently with 1.99 points, we find the strategy of Catastrophizing which to a lesser extent leads young people to focus their thoughts on serious consequences in a disproportionate way. Finally, the least frequent cognitive strategy is that of Self-blame with 1.40 points, the closest to the term “never” by which some young people hardly ever take care of the current situation of compulsory isolation and university closure due to the pandemic.

We have carried out a comparison between averages to investigate possible differences between the Spanish student population, with 1566 subjects, and the non-Spanish student population, in which we grouped the 344 subjects from different Latin American universities.

Table 6 shows that the values of the means are very similar for the variables Self-blame with a Spanish mean of 1.69 and a Latin American mean of 1.90; Acceptance with a Spanish mean of 3.75 and a Latin American mean of 3.68; Rumination with a Spanish mean of 3.35 and a Latin American mean of 3.04; Positive refocusing with a Spanish mean of 3.34 and a Latin American mean of 3.59; Refocus on planning with Spanish average of 3.45 and a Latin American average of 3.52; Positive reappraisal with a Spanish average of 3.66 and a Latin American average of 3.89; Putting into perspective with a Spanish average of 3.81 and a Latin American average of 3.71; Catastrophizing with a Spanish average of 2.35 and a Latin American average of 2.36; and Other-blame with a Spanish mean of 2.86 and a Latin American mean of 2.89.

**TABLE 6:** Comparison between averages.

**Strategy**	**Population**	***N***	**$\bar{X}$**	**DS**	**Typical average error**
Self-blame	Spanish	1566	1.69	0.632	0.016
	Latinoamerican	344	1.90	0.658	0.035
Acceptance	Spanish	1566	3.75	0.854	0.022
	Latinoamerican	344	3.68	0.852	0.046
Rumination	Spanish	1566	3.35	1.051	0.027
	Latinoamerican	344	3.04	1.031	0.056
Positive refocusing	Spanish	1566	3.34	1.129	0.029
	Latinoamerican	344	3.59	1.018	0.055
Refocus on planning	Spanish	1566	3.45	0.928	0.023
	Latinoamerican	344	3.52	0.922	0.050
Positive reappraisal	Spanish	1566	3.66	1.066	0.027
	Latinoamerican	344	3.89	0.994	0.054
Putting into perspective	Spanish	1566	3.81	0.913	0.023
	Latinoamerican	344	3.71	0.899	0.048
Catastrophizing	Spanish	1566	2.35	0.862	0.022
	Latinoamerican	344	2.36	0.846	0.046
Other-blame	Spanish	1566	2.86	1.237	0.031
	Latinoamerican	344	2.89	1.162	0.063

However, as shown in Table 7, when the non-parametric Mann–Whitney test is applied to independent samples (because it does not meet the condition of normality for parametric contrast), the result shows significant values in the Self-blame, Positive refocusing, Positive reappraisal variables in favor of the Latin American population; and in the Acceptance, Rumination and Putting into perspective variables in favor of the Spanish population. In both populations, two different adaptive strategies predominate, Positive refocusing and Positive reappraisal, as opposed to Acceptance and Putting into perspective. And in both populations, they differ in the predominance of a different non-adaptive strategy: Self-blame versus Rumination.

**TABLE 7:** Mann–Whitney test.

**Estrategies**	**U de Mann–whitney**	**W de wilcoxon**	***z***	**Bil. A. Sig.**
Self-blame	222667.500	1449628.500	−5.678	0.000
Acceptance	251629.000	310969.000	−2.053	0.040
Rumination	223841.000	283181.000	−5.096	0.000
Positive refocusing	237213.500	1464174.500	−3.582	0.000
Refocus on planning	259448.000	1486409.000	−1.126	0.260
Positive reappraisa	238398.500	1465359.500	−3.469	0.001
Putting into perspective	250881.000	310221.000	−2.100	0.036
Catastrophizing	265449.000	1492410.000	−0.459	0.646
Other-blame	263243.500	1490204.500	−0.681	0.496

### CFA (Structural Equation Model)

The CFA is used to confirm the structure and measurement capability of the dimensions that make up the CERQ. The Path Analysis represents another way to estimate the relationship between variables that are directly observed. The Structural Equation Modeling -SEM- provides the most solid procedures and technical criteria for the validation of measurement models under these two assumptions. This is the analysis we have carried out.

**FIGURE 2:**
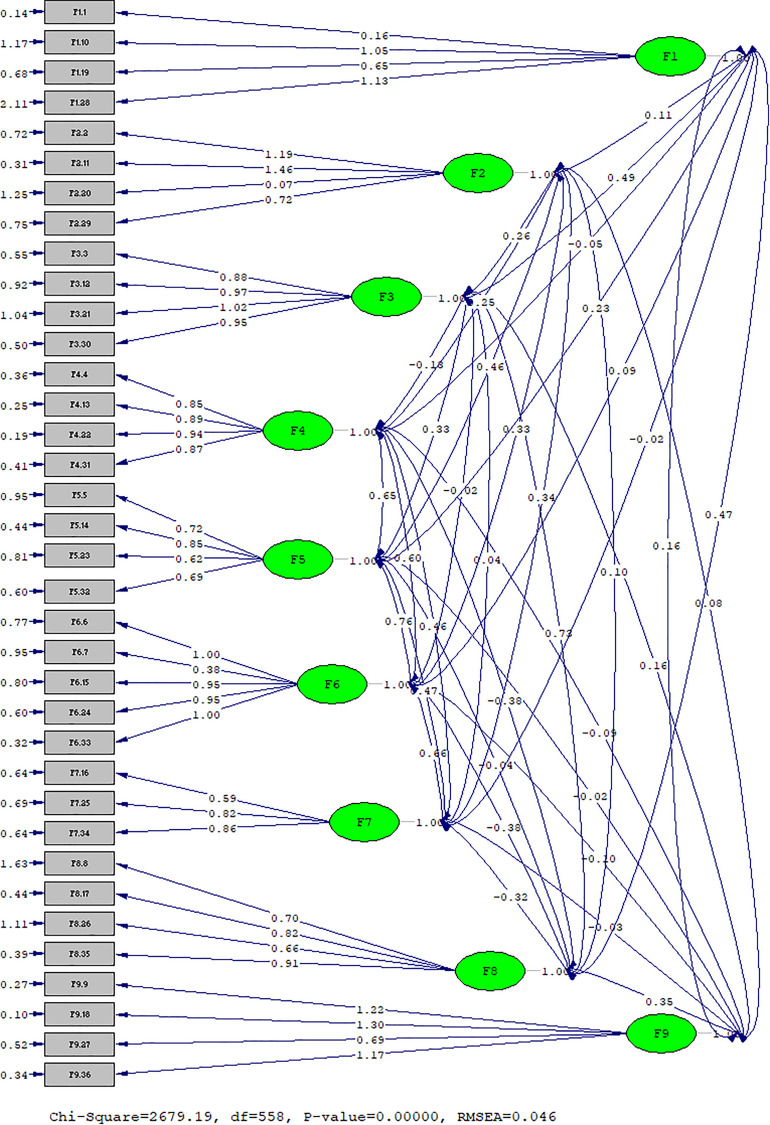
Graphical representation of the natural measurement model of the Likert scale at CERQ.

We estimated the parameters of the model found based on the graphical representation that appears in Figure 2.

In Dimension 1, Self-blame, the belief that *the causes of isolation correspond to me* (F1.28) with 1.13 is the idea that has more weight, as opposed to *feeling the only person guilty for this situation* (F1.1) with 0.16 that reaches the lowest weight. On dimension 2, Acceptance, the idea given the highest weight is *I have to accept the situation* (F2.11) with 1.46 and the idea with the lowest weight is the belief *I cannot change anything about this situation* (F2.20) with 0.07. As for dimension 3, Rumination, the item that scores best is *willing to understand why isolation makes me feel this way* (F2.21) with 1.02 and the item with less weight is *to stop thinking about how I feel about the isolation* (F3.3) with 0.88. Regarding dimension 4, Positive Refocusing, the most important idea is *to think about pleasant things instead of lockdown situation* (F4.22) with 0.94, meanwhile *thinking about things more pleasant things* has the lowest score with 0.85 being the less important item. On dimension 5, Refocus on Planning, the item with the greatest importance is *to think about how best to deal with the situation* (F5.14) with 0.85 being the less relevant *how the situation could change* (F5.23) with 0.62. When analyzing dimension 6, Positive Reappraisal, with five items instead of 4, the items *I think I can learn something from this situation* (F6.6) and to *look for the positive aspects of the situation* (F6.33) both with 1.00 are the two ideas with more weight, as opposed to *everything could have been much worse* (F6. 7) with 0.38) the one with less weight, an item that in the original structure belongs to dimension 7 Putting into Perspective and that in our model fits into dimension 6. Therefore, our dimension 7, Putting into Perspective, is left with only three items among which it appears that the idea with more weight is *there are worse things in life* (F7.34) with 0.86, as opposed to *I think other people go through worse experiences* (F7.16) with 0.59. On the other hand, in dimension 8, Catastrophizing, we find that the item with the highest weight is *thinking continuously about how horrible this situation is* (F8.35) with 0.91 and the item with the lowest weight is *this is the worst thing that could happen to a person* (F8.26) with 0.66. Finally, in dimension 9, Other-blame, the most relevant idea is that *others are responsible for what is happening* (F9.18) with 1.30 versus the less important *to feed in the errors that others have made in this matter* (F9.27) with 0.69.

The dimensions Refocus on Planning (F5), and Positive Reappraisal (F6) with 0.76 show a very strong relationship among them. On the other hand, the dimensions Rumination (F3) with Catastrophizing (F8) with 0.73, and Positive Refocusing (F4) with Refocus on Planning (F5) with a ratio of 0.65, also show a strong relationship to each other.

The main inverse relationships between dimensions are between Catastrophizing (F8) and three others: Positive Reappraisal (F6) with −0.38; Positive Refocusing (F4) with −0.38; and Putting into Perspective (F7) with −0.32.

We can say that for the students surveyed the Refocus on Planning is greatly influenced by the Positive Reappraisal and Positive Refocusing of the problem. In addition, that an Excessive Reflection (Rumination) on stressful issues generates Catastrophizing.

The structural equation model obtained, converges with an excellent fit and we consider it confirmed. The goodness indices (Lévy and Varela, 2006) are included in Table 8.

**TABLE 8:** Goodness-of-fit indices.

	**X^2^**	**X^2^/gl**	**GFI**	**RMSEA**	**ECVI**	**IFI**	**NFI**	**RFI**
Real value	*p* = 0.000	4.80	0.90	0.046	0.74	0.97	0.96	0.96
Ideal value	*p* < 5	<5	>0.90	<0.08	>valor	>0.95	>0.95	>0.95

## Discussion

Numerous researchers have used the CERQ scale on university students to clarify various aspects of their psychological well-being, social adjustment and emotional regulation (Martin and Dahlen, 2005; Abdi et al., 2012; Tuna and Bozo, 2012; Castro et al., 2013; Medrano et al., 2013; Costa Martins et al., 2016; Dominguez and Medrano, 2016). In this research, the CERQ instrument has proven to be effective in characterizing coping strategies for young students. Therefore, we understand that the instrument can be very useful in educational research to know emotional aspects linked to learning in Higher Education and to academic success or failure. In situations of virtual teaching and e-learning, in which the face-to-face teacher-student relationship is weakened, it will be especially useful.

We have seen how the goodness adjustment of the CERQ model presented, confirms the original structure in 9 dimensions (GFI,90 and RMSEA,046) generated by Garnefski and Kraaij (2007) as the studies in its Spanish application by Domínguez-Sánchez et al. (2013); Reche (2019) or Chamizo-Nieto et al. (2020) among others do. In any case, we have found a mismatch with respect to the original dimensionalization in an item that goes from dimension F7 to dimension F6, that is, from Putting into Perspective to Positive Reappraisal. These small mismatches have been found in other studies such as that of Medrano et al. (2013) applied in Spanish language to a population of Argentine university students or in that of Jermann et al. (2006) or Abdi et al. (2012) which refer to the low weight or negative weight of some item in the Catastrophizing dimension. We have not found any study that refers to our finding regarding item 7: *I think everything could have been much worse.*

The results obtained with the application of the CERQ differ in the different studies depending on the sample (age or composition) and the target (general or specific situation). Thus, in the original by Garnefski and Kraaij (2007), Catastrophizing and Rumination appear as two of the most used strategies. In Reche’s study (2019) with fibromyalgia patients, the results are more similar to ours since the most used cognitive strategies are the most adaptive ones as opposed to the less adaptive ones such as Catastrophizing, Self-blame and Other-blame which reach lower frequencies. Perhaps the similarity of results is due to the fact that in both cases, the instrument was given a specific use (illness, in the case of Reche, and lockdown, in ours) and the cognitive strains for emotional regulation differ when referred to a very specific stressful event, as opposed to coping with general stress, which is how it is used in other studies.

The cognitive strategies used by the students surveyed have allowed them coping with events arising from the pandemic, mandatory isolation and university closure, certainly adaptive and functional, while keeping a positive perception of their new living and learning situation. Fiorillo and Gorwood (2020), as well as Holmes et al. (2020) have pointed out that the consequences on the mental health and psychological well-being of the general population will increase over the weeks with effects similar to those generated by such stressful catastrophic events as natural disasters, earthquakes or tsunamis, or even wars and major international conflicts. Even so, from the perspective of Pfefferbaum and North (2020), the consequences of this pandemic do not currently meet the criteria for consideration as PTSD (post-traumatic stress disorder).

However, out of the population in direct contact with the virus (sick people with their families and caregivers and health workers), the general population suffers the stress of their daily activities closure, mandatory lockdown and overexposure to news about the pandemic. We agree with Fiorillo and Gorwood (2020) that the consequences take some time to be visible and therefore we consider our study, carried out during the first 6 weeks of home isolation, as a baseline in which there are still no evident signs of emotional distress in the population studied, and which reveals that the Catastrophizing strategy as a means of emotional regulation is one of the least used by young people. It is a strategy which, in this case, is not functional (Dominguez and Medrano, 2016) because its warning capacity does not allow to stay away from danger once physical-social distance measures and home isolation have been established. Holmes et al. (2020) warn that physical distance or social isolation will produce negative feelings in the general population which are at the root of anxiety, stress and depression. Cognitive coping strategies are used to minimize these risks. In the population studied, we have not yet seen signs of serious consequences since coping is carried out mainly by Putting into Perspective, Acceptance, Positive Reinterpretation, Refocus on Planning and Positive Refocusing as these are very adaptive strategies that move away from the bias of Self-blame, an indicator of depressive effects (Medrano et al., 2013) in emotional regulation. As Holmes et al. (2020) point out, the social isolation that is compensated by the strengthening of family ties, due to forced social distancing, is mitigating the negative effect and generating resilience to diminish the stressful circumstances. In our study, most young people are being confined to family homes in an average group of 3 or 4 people and in housing conditions that are considered optimal for the vast majority. This is consistent with the effective coping style they are following, at least in the first weeks of lockdown.

The data we have from China, the first region in the world to declare a pandemic state, were collected in February 2020, 3 months after the recognition of the covid-19 infection by health authorities. These data from the population with longer exposure time to the pandemic, does show the increase in indicators of emotional distress such as anxiety, depression, indignation or unhappiness, as shown by Li et al. (2020) in their analysis of thousands of posts made in the largest social network in the country. These data suggest that, despite the positive coping the students we have surveyed are doing at the moment, they may be faced with pernicious effects on their psychological well-being as the weeks go by. Holmes et al. (2020); Pfefferbaum and North (2020) all point out that the effect of the coming economic crisis, in Western Europe and Latin America, and which will affect the less affluent population, will contribute to the worsening of mental health. The study by Wang et al. (2020), completed in February 2020, already indicates that more than half of the sample -1210 Chinese citizens- has a moderate to severe negative psychological impact (16.5% with depressive symptoms). Although our study does not reveal this, we fear that this is something that in other regions where the pandemic has come later, may be yet to come.

Van Bavel et al. (2020) draw the attention to the emotional effect generated by the feeling of threat and the optimism bias. They find that the threats transmitted by health authorities to produce cautious reactions in the population are only valid if people can opt for protective behavior. Otherwise, people generate self-defensive feelings to justify their own inaction. In our study we have seen how young people are aware of the real threat of the pandemic, of their own sensitivity to contagion and of their role as a vector of contagion. That is, they are well informed and aware and have not opted for defensive emotions or those related to optimism bias that would have led to the underestimation of risk. One of the results of our study is the self-evaluation the students show about their digital competence and their capacity to develop in the virtual communicative interaction. For Van Bavel et al. (2020) this is key to rejecting a feeling of loneliness or social isolation, even if there is momentary physical separation from friends and classmates [as it has been previously studied with school populations in homebound due to illness or disability by Benigno et al. (2014) and Trentin et al. (2015)] which is consistent with the results of emotional well-being students surveyed present. The university students analyzed have had to seriously modify their study habits to adapt to a new teaching model. They have transformed the consumption of several hours a week of listening to their teachers’ lectures into more interactive learning strategies centered on the search, selection and reading of documentation, the carrying out of open tasks and the provision of evidence of the learning undertaken in order to obtain an evaluation. This would not have been possible without that feeling of optimism, positivity, confidence in their digital skills and social support maintained through the networks, as we have pointed out.

## Data Availability Statement

The raw data supporting the conclusions of this article will be made available by the authors, without undue reservation.

## Ethics Statement

Ethical review and approval was not required for the study on human participants in accordance with the local legislation and institutional requirements. The patients/participants provided their written informed consent to participate in this study.

## Author Contributions

JAR, MCL, and FDR have carried out literature review and the design of the studio. IAR, EGC, PIC, and EJLS have selected evaluation instruments, developed the first version of the specific instrument and directed the application of both. dBCC has coordinated the international application. DGG and AHF have performed the data analysis. MFC has coordinated the study. All authors contributed to the article and approved the submitted version.

## Conflict of Interest

The authors declare that the research was conducted in the absence of any commercial or financial relationships that could be construed as a potential conflict of interest.

## References

[B1] Abdi S. Taban S. Ghaemian A. (2012). Cognitive emotion regulation questionnaire: validity and reliability of Persian translation of CERQ-36 item. *Proc. Soc. Behav. Sci.* 32 2–7. 10.1016/j.sbspro.2012.01.001

[B2] Aulck L. Velagapudi N. Blumenstock J. West J. (2017). Predicting student dropout in higher education. *arXiv* [Preprint]. 1606.06364.

[B3] Ávila Quiñones A. S. Montaña G. J. Jiménez Arenas D. Burgos J. P. (2014). Estilos y estrategias de afrontamiento y rendimiento académico: una revisión empírica. *Enfoques* 1 15–44. 10.24267/23898798.79

[B4] Bar-Am R. Arar O. (2017). Dropouts and budgets: a test of a dropout reduction model among students in israeli higher education. *Eur. J. Educ. Res.* 6 123–134. 10.12973/eu-jer.6.2.134

[B5] Benigno V. Caruso G. Ravicchio F. Repetto M. Trentin G. (2014). Il progetto TRIS e l’inclusione socio-educativa degli studenti impossibilitati alla normale frequenza scolastica. *Bisog. Educ. Spec. Prat. Inclus.* 14 1–14.

[B6] Bolhaar J. Gerritsen S. Kuijpers S. van der Wiel K. (2019). *Experimenting With Dropout Prevention Policies (No. 400. rdf).* Hague: CPB Netherlands Bureau for Economic Policy Analysis.

[B7] Castro J. Chaves B. Pereira A. T. Soares M. J. Amaral A. P. Bos S. (2013). Cognitive emotions regulation questionnaire: validation of the portuguese versión. *Atenc. Primar.* 45:162.

[B8] Chamizo-Nieto M. T. Rey L. Sánchez-Álvarez N. (2020). Validation of the spanish version of the cognitive emotion regulation questionnaire in adolescents. *Psicothema* 32 153–159. 10.7334/psicothema2019.156 31954429

[B9] Costa Martins E. Freire M. Ferreira-Santos F. (2016). Examination of adaptive and maladaptive cognitive emotion regulation strategies as transdiagnostic processes: associations with diverse psychological symptoms in college students. *Stud. Psychol.* 58 59–73. 10.21909/sp.2016.01.707

[B10] Dominguez S. Medrano L. (2016). Propiedades psicométricas del Cognitive Emotional Regulation Questionnarie (CERQ) en estudiantes universitarios de Lima. *Psychologia* 10 53–67. 10.21500/19002386.2466

[B11] Domínguez-Sánchez F. J. Lasa-Aristu A. Amor P. J. Holgado-Tello F. P. (2013). Psychometric properties of the spanish version of the cognitive emotion regulation questionnaire. *Assessment* 20 253–261. 10.1177/1073191110397274 21467092

[B12] Fernández C. M. (2015). *Formación y Desarrollo de Profesionales de la Educación: Un Enfoque Profund*o. Blue Mounds, WI: Deep University Press.

[B13] Fiorillo A. Gorwood P. (2020). The consequences of the COVID-19 pandemic on mental health and implications for clinical practice. *Eur. Psychiatry* 20 1–4. 10.1192/j.eurpsy.2020.35 32234102 PMC7156565

[B14] Garnefski N. Kraaij V. (2007). The cognitive emotion regulation questionnaire. *Eur. J. Psychol. Assessment* 23 141–149. 10.1027/1015-5759.23.3.141

[B15] Garnefski N. Kraaij V. Spinhoven P. (2001). Negative life events, cognitive emotion regulation and emotional problems. *Pers. Individ. Differ.* 30 1311–1327. 10.1016/S0191-8869(00)00113-6

[B16] Gross J. J. (2015). Emotion refgulation: current status and future prospect. *Psychol. Inq.* 26 1–26. 10.1080/1047840X.2014.940781

[B17] Holmes E. A. O’Connor R. C. Perry V. H. Tracey I. Wessely S. Arseneault L. (2020). Multidisciplinary research priorities for the COVID-19 pandemic: a call for action for mental health science. *Lancet Psychiatry* 7 547–560. 10.1016/S2215-0366(20)30168-1 32304649 PMC7159850

[B18] Jermann F. Van der Linden M. d’Acremont M. Zermatten A. (2006). Cognitive emotion regulation questionnaire (CERQ). Confirmatory factor analisys and Psychometric Properties of the French Translation. *Eur. J. Psychol. Assessment* 22 126–131. 10.1027/1015-5759.22.2.126

[B19] Judge L. Rahman F. (2020). *Lockdown Living: Housing Quality Across the Generations.* Westminster: Resolution Foundation, Corp Creator.

[B20] Lévy J. P. Varela J. (2006). *Modelización con Estructuras de Covarianzas en Ciencias Sociales: Temas Esenciales, Avanzados y Aportaciones Especiales.* Coruña: Netbiblo.

[B21] Li S. Wang Y. Xue J. Zhao N. Zhu T. (2020). The impact of COVID-19 epidemic declaration on psychological consequences: a study on active Weibo users. *Int. J. Environ. Res. Public Health* 17:2032. 10.3390/ijerph17062032 32204411 PMC7143846

[B22] Lizarte E. J. (2017). *Análisis del Abandono de los Estudios en la Universidad de Granada. El caso de la Facultad de Ciencias de la Educación.* Ph.D. Tesis, Universidad de Granada, Granada.

[B23] Majumdar P. Biswas A. Sahu S. (2020). COVID-19 pandemic and lockdown: cause of sleep disruption, depression, somatic pain, and increased screen exposure of office workers and students of India. *Chronobiol. Int.* 20 1–10. 10.1080/07420528.2020.1786107 32660352

[B24] Martin R. C. Dahlen E. R. (2005). Cognitive emotion regulation in the prediction of depression, anxiety, stress and anger. *Pers. Individ. Differ.* 39 1249–1260. 10.1016/j.paid.2005.06.004

[B25] Martínez-Sánchez I. Goig-Martínez R. M. Álvarez-Rodríguez J. Fernández-Cruz M. (2020). Factors contributing to mobile phone dependence amongst young people—educational implications. *Sustainability* 12:2554 10.3390/su12062554

[B26] McRae K. (2016). Cognitive emotion regulation: a review of theory and scientific findings. *Curr. Opin. Behav. Sci.* 10 119–124. 10.1016/j.cobeha.2016.06.004

[B27] Medrano L. A. Moretti L. Ortiz A. Pereno G. (2013). Validación del cuestionario de regulación emocional cognitiva en universitarios de córdoba, argentina. *Psykhe* 22 83–96. 10.7764/psykhe.22.1.473

[B28] Morales M. Trianes M. (2010). Estrategias de Afrontamiento e Inadaptación en niños y adolescentes. *Eur. J. Educ. Psychol.* 3 275–286. 10.30552/ejep.v3i2.42

[B29] Mortagy Y. Boghikian-Whitby S. Helou I. (2018). An analytical investigation of the characteristics of the dropout students in higher education. *Issues Informing Sci. Inform. Technol.* 15 249–278. 10.28945/3999

[B30] Pfefferbaum B. North C. S. (2020). Mental health and the Covid-19 pandemic. *New Engl. J. Med.* 20:8017. 10.1056/NEJMp2008017 32283003

[B31] Reche C. E. (2019). *Evaluación de la Regulación Emocional Cognitiva en Fibromialgia Mediante el Instrumento CERQ.* Ph.D. Tesis, Universidad Autónoma de Barcelona, Barcelona.

[B32] Rockinson-Szapkiw A. J. Holmes J. Stephens J. S. (2019). Identifying significant personal and program factors that predict online EdD Students’ program integration. *Online Learn.* 23:1579 10.24059/olj.v23i4.1579

[B33] Shea P. Bidjerano T. (2019). Effects of online course load on degree completion, transfer, and dropout among community college students of the state university of New York. *Online Learn.* 23:1364 10.24059/olj.v23i4.1364

[B34] Singh S. Roy M. D. Sinha C. P. T. M. K. Parveen C. P. T. M. S. Sharma C. P. T. G. Joshi C. P. T. G. (2020). Impact of COVID-19 and lockdown on mental health of children and adolescents: a narrative review with recommendations. *Psychiatry Res.* 20:113429. 10.1016/j.psychres.2020.113429 32882598 PMC7444649

[B35] Stikkelbroek Y. Bodden D. H. Kleinjan M. Reijnders M. van Baar A. L. (2016). Adolescent depression and negative life events, the mediating role of cognitive emotion regulation. *PLoS One* 11:1062. 10.1371/journal.pone.0161062 27571274 PMC5003336

[B36] Tejedor F. García A. Muñoz V. (2007). Causas del bajo rendimiento del estudiante universitario (en opinión de los profesores y alumnos). Propuestas de mejora en el marco del EEES. *Rev. Educ.* 342 443–473.

[B37] Trentin G. Benigno V. Caruso G. Ravicchio F. Repetto M. (2015). Hybrid Learning Spaces for the socioeducational inclusion of homebound students. *Proc. IICE* 15 20–22. 10.20533/ijtie.2047.0533.2015.0088

[B38] Tuna E. Bozo Ö (2012). The cognitive emotion regulation questionnaire: factor structure and psychometric properties of the turkish version. *J. Psychopathol. Behav. Assess.* 34 564–570. 10.1007/s10862-012-9303-8

[B39] Van Bavel J. J. Baicker K. Boggio P. S. Capraro V. Cichocka A. Cikara M. (2020). Using social and behavioural science to support COVID-19 pandemic response. *Nat. Hum. Behav.* 20 1–12. 10.1038/s41562-020-0884-z 32355299

[B40] Wang C. Pan R. Wan X. Tan Y. Xu L. Ho C. S. (2020). Immediate psychological responses and associated factors during the initial stage of the 2019 coronavirus disease (COVID-19) epidemic among the general population in China. *Int. J. Environ. Res. Public Health* 17:1729. 10.3390/ijerph17051729 32155789 PMC7084952

